# Beneficial effects of alpha-1 antitrypsin therapy in a mouse model of colitis-associated colon cancer

**DOI:** 10.1186/s12885-023-11195-5

**Published:** 2023-08-02

**Authors:** Mariam Al-Omari, Tareq Al-Omari, Nesreen Batainah, Khaled Al-Qauod, Beata Olejnicka, Sabina Janciauskiene

**Affiliations:** 1 Department of Basic Medical Sciences, Faculty of Medicine, Yarmouk University, P.O Box 566, Irbid, 21163 Jordan; 2 Department of Biological Sciences, Faculty of Science, Yarmouk University, Irbid, Jordan; 3 Department of Pulmonary and Infectious Diseases and BREATH German Center for Lung Research (DZL), Hannover Medical School, Hannover, Germany; 4 Department of Internal Medicine, Lund University, Skåne University Hospital Malmö, Malmö, Sweden

**Keywords:** Colorectal cancer, Mice model, Alpha1-antitrypsin, Inflammation, Neutrophils, Caspase-3, Granzyme-B, Cytokines, Matrix metalloproteinases

## Abstract

**Background:**

It is widely accepted that chronic inflammatory bowel diseases significantly higher a risk for colorectal cancer development. Among different types of treatments for patients with colon cancer, novel protein-based therapeutic strategies are considered.

**AIM:**

To explore the effect of human plasma alpha-1 antitrypsin (AAT) protein in the chemically induced mouse model of colorectal cancer.

**Methods:**

BALB/c mice with azoxymethane/dextran sodium sulfate (AOM/DSS)-induced colitis-associated colorectal cancer (CAC), we intraperitoneally treated with commercial preparation of human plasma AAT (4 mg per mouse). Effects of this therapy were evaluated histologically, and by immunohistochemical and gene expression assays.

**Results:**

When compared with non-treated controls, AOM/DSS mice receiving AAT therapy exhibited significantly longer colons, and less anal bleeding. Concurrently, AAT-treated mice had significantly fewer polyps, and lower numbers of large colon tumors. Immunohistochemical examinations of colon tissues showed significantly lower neutrophil counts, more granzyme B-positive but fewer MMP9 (gelatinase B)-positive cancer cells and lower numbers of apoptotic cells in mice receiving AAT therapy. The expression levels of *IL4* were significantly higher while *TNFA* was slightly reduced in tumor tissues of AOM/DSS mice treated with AAT than in AOM/DSS mice.

**Conclusion:**

Human AAT is an acute phase protein with a broad-protease inhibitory and immunomodulatory activities used as a therapeutic for emphysema patients with inherited AAT deficiency. Our results are consistent with previous findings and support an idea that AAT alone and/or in combination with available anti-cancer therapies may represent a new personalized approach for patients with colitis-induced colon cancer.

**Supplementary Information:**

The online version contains supplementary material available at 10.1186/s12885-023-11195-5.

## Introduction

Colorectal cancer (CRC) includes colon and/or rectum cancer, and is the third most commonly diagnosed and second most fatal cancer globally [1]. For example, in Jordan, CRC ranked as most common in males and females accounting for about 12.2% of all cancers [2]. In Germany, CRC accounts for about 60 000 cases and 25 000 deaths per year [3].

CRC typically develops when epithelial cells acquire a series of genetic or epigenetic changes enabling them to become hyperproliferative [4]. The increased risk for CRC is strongly associated with a family history. As an example, a large-scale meta-analysis of 8091 subjects found that the mean risk is almost twice higher in those with a family history of CRC [5]. On the other hand, dietary, lifestyle, anthropometric, and other risk factors also play a significant role in CRC development [6].

Surgery is the most common treatment for CRC patients. However, many CRC cases are diagnosed at the advanced stage, and therefore, curative surgery alone is often challenging [7]. To stabilize the tumor, chemotherapy or radiotherapy is used before the surgery [8]. In recent years, an increasing number of monotherapy or combination therapy strategies using immune checkpoint inhibitors for CRC have been designed [9]. Cytokines, like TNF-α and IL-6, are also important drivers of CRC development and are therapeutic targets [10].

Research also focuses on natural products, such as alkaloids, polysaccharides, polyphenols, diterpenoids, and unsaturated fatty acids, as useful therapeutics for CRC prevention and/or treatment [11]. For example, case-controlled studies revealed an inverse correlation between the levels of vitamin D and the incidence of human CRC [12]. Among the mechanisms proposed to explain this association are the immunomodulatory, antiangiogenetic, and pro-apoptotic effects of vitamin D [13]. Scientists are testing natural and/or recombinant human protein therapies as well. Under physiological conditions, various proteins control cell growth, survival, and responses to stimuli. Therefore, the deregulation of levels and/or functional activities of certain proteins can favor the development of cancer [14]. Among others, acute-phase proteins are involved in cancer development and some of them considered as therapeutics in CRC [15, 16].

Human AAT is a serine protease inhibitor and one of the most abundant acute-phase glycoproteins (1–2 g/L) expressing broad protease inhibitory and immunomodulatory effects [16, 17]. It is known that inherited AAT deficiency is a risk factor for developing early onset emphysema, liver disease at any age, and in some cases panniculitis and systemic vasculitis [18, 19]. Various clinical studies reported that AAT deficiency is also associated with hepatocellular carcinomas [20–22], lung cancer [23, 24], urinary bladder cancer [25], and malignant lymphomas [26]. As a matter of fact, both high and low levels of AAT have been linked to the development of CRC [20, 27–29], and AAT has been proposed as a biomarker for detecting early stages of gastrointestinal inflammation [30]. Though the relationship between AAT and CRC remains controversial, the fact that AAT deficiency is linked to various cancers, and that the therapy with AAT expresses anti-inflammatory effects [31] prompted us to test the effects of AAT therapy in a mouse model of colitis-associated cancer (CAC). The CAC is a subtype of CRC that is associated with inflammatory bowel diseases and has a high mortality. A mouse model based on the combination of a colonic genotoxic carcinogen, azoxymethane (AOM), and an inducer of colitis, dextran sulfate sodium (DSS), represents one of the best tools for investigating the pathogenesis and/or prevention of colon cancer [32]. In this study, we used a model of AOM/DSS-induced CAC in BALB/c mouse to evaluate the effects of an intraperitoneally administered commercial preparation of human AAT (Prolastin).

## Materials and methods

### Animal model

Male BALB/c mice aged 8 weeks and weighing 27 g were provided by the animal facility, Yarmouk University, Irbid, Jordan. The animal groups were housed separately in plastic cages and received a normal diet and water ad libitum, with a light/dark cycle of 12:12 h. Food and water consumption were comparable between the treated and control groups. All measures were taken to avoid unnecessary animal stresses. Housing, anesthesia, and postoperative care concurred with the guidelines established by an Institutional Animal Ethics Committee approval (ACU-2021/11) Yarmouk University.

**Fig. 1:**
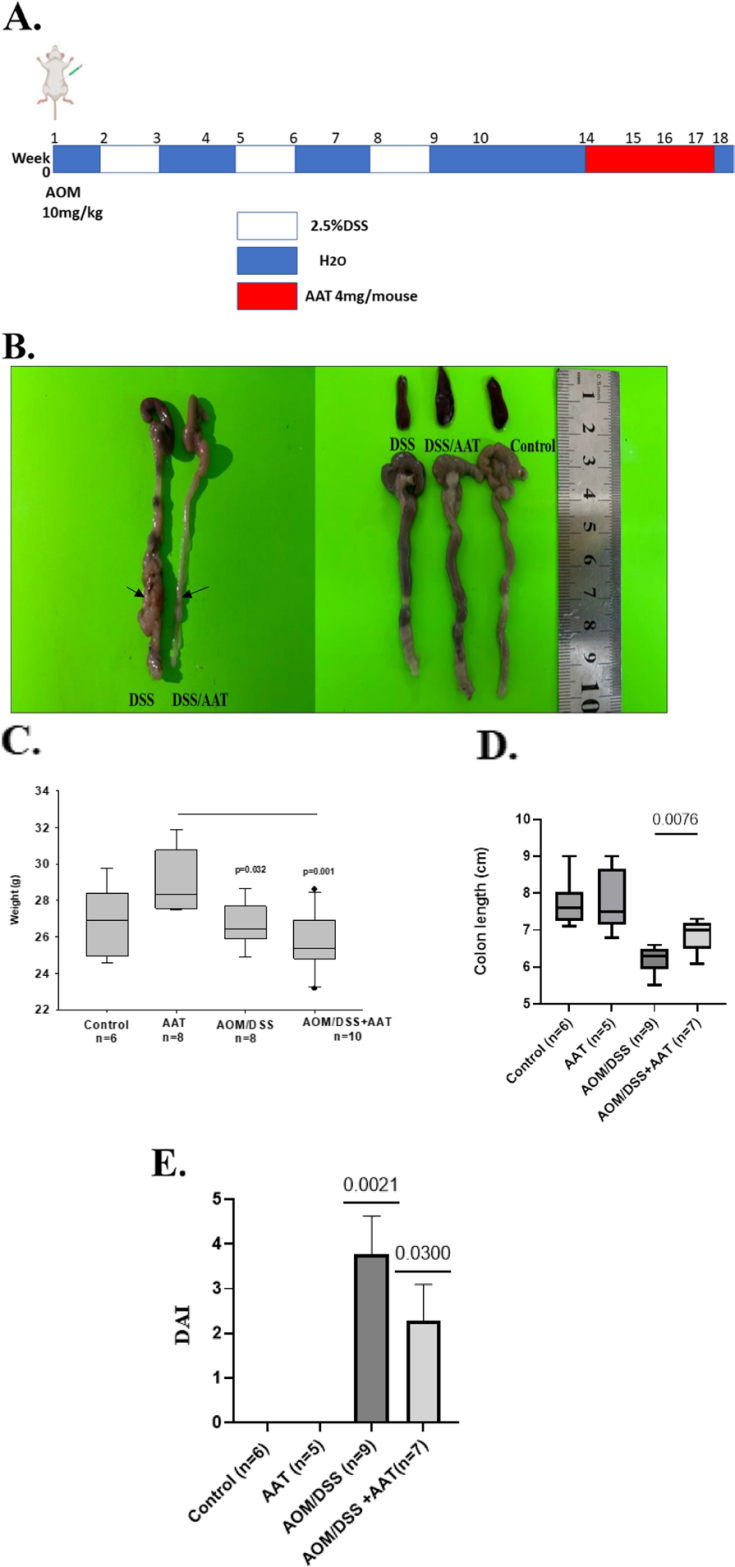
Schematic presentation of study design and AAT therapy effects on mice colon length and body weight. **A**. Mice were injected with AOM (day 0) followed by three cycles of 2.5% DSS in drinking water. AOM/DSS-AAT group received i.p. AAT on weeks 15, 16 and 17. At week 18, all mice were sacrificed for further analysis. **B**. Representative gross macroscopic image of the colon of AOM/DSS and AOM/DSS + AAT. **C.** Box plots show body weight differences between experimental groups of mice. **D**. Colon length, box plots show colon length in different experimental groups. **E**. Disease Activity Index (DAI) score.a composite measure of weight loss, stool consistency and blood in stool. A student’s t-test was used to compare between each two groups. A value of *p* < 0.05 was considered a significant

The AOM/DSS model [33] was based on a single intraperitoneal injection of (10 mg/kg body weight) AOM (ChemCruze, USA) and three cycles of 2.5% of DSS (TdB Consultancy, Sweden) in drinking water, over a period of ten weeks. Mice were randomly divided into four groups (*n* = 10 mice per group); group 1 was the controls receiving water; group 2 received AOM/DSS only, group 3 received AOM/DSS and was injected intraperitoneally with human AAT protein (Grifols, Germany) and the group 4 received AAT only. Mice were injected with 4 mg of AAT at 15, 16, and 17 weeks. A schematic design of the experiment is shown in Fig. 1A.

Animals were individually weighed on a weekly basis and signs of bloody stool and diarrhea were recorded. On the day of experiment termination (end of week 17), the colon was recovered from each mouse and inspected for the presence of abnormalities, including tumor masses, size, and other deformations. Colon length and weight were measured, and tissues were collected for RNA extraction and gross analyses.

### Histological examination

Mice colon tissues were fixed in 10% formalin for 24 h, and cassetted. Tissue processing was performed using Spin Tissue Processor (Thermo Scientific, USA). Samples were serially dehydrated in baths with increasing ethanol percentage (70%, 80%, 96%, and 100%) for approximately 1.5 h each. Subsequently, tissue samples were placed in 100% xylene for 3 h to allow for tissue clearing. Samples were then incubated in paraffin wax for 4 h. After that, samples were placed into a mold maintaining their original orientation, embedded in 100% paraffin wax, and then left to cool at room temperature (RT) overnight. Paraffin blocks were cut into thin Sects. (5 µm) using Electronic Rotary Microtome (Thermo Scientific, USA). Sections were stained with hematoxylin and eosin (H&E) and slides were evaluated microscopically [34].

### Immunohistochemistry

Mice colonic sections were harvested and fixed in 10% (v/v) formalin. Paraffin-embedded tissues were cut (5 µm) and fixed on slides coated with poly-L lysine. After being deparaffinized in xylene and rehydrated in graded alcohol, the formalin-fixed paraffin-embedded specimens were treated with boiling in 10 mmol/L citrate buffer (pH 6.0) for 1 min using the microwave. Afterwards samples were cooled to RT and washed 3 times with phosphate-buffered saline (PBS). The next steps were carried out by using an immunohistochemistry kit (Abcam, UK). Endogenous peroxidase activity was blocked using 3% hydrogen peroxide for 15 min, and after washing the sections were blocked with 1% BSA in PBS for 10 min. All primary antibodies were rabbit polyclonal anti-AAT (DAKO Denmark), anti-MMP9 (MyBiosource, USA) anti-Granzyme-B (ELK Biotechnology, China), and anti-Caspase-3 (CUSABIO, USA) were diluted according to the manufacturer's recommendations and added to tissue sections for 1 h. After washing, slides were incubated with anti-rabbit-HRP (Promega, USA) at RT for 15 min. Tissues were covered with Diaminobenzidine (DAB) Substrate (Bioworld, USA) according to the manufacturer’s instructions. All sections were counterstained with Hematoxylin and rinsed with d.H2O, ascendingly dehydrated in graded alcohols followed by xylene. Then mounted with DPX and visualized under a light microscope. Grading was done based on the intensity and the extent of positivity as scored by an experienced pathologist.

### Detection of apoptosis by TUNEL assay

The TUNEL assay (Promega, USA) was performed after pre-treatment of paraffin-embedded tissue. Briefly, five μm tissue sections were layered to poly-L-lysine-coated slides. Subsequently, sections were deparaffinized and rehydrated. Following that, tissue sections were fixed with 4% paraformaldehyde in two separate steps, Protein digestion was done by incubating tissue sections in 20 mg/ml proteinase K (Promega, USA) for 10 min at RT. A rinsing step with PBS was carried out between each step of the experiment. Tissue sections were equilibrated in an equilibration buffer for 10 min at RT. The labeling mixture, which included biotinylated dUTP and rTdT enzyme in equilibrating buffer, was applied to sections and incubated for 60 min at 37 °C in a humid chamber. After stopping the enzymatic reaction by immersing tissue sections in 2X SSC solutions for 15 min, the sections were rinsed thoroughly twice with PBS, and endogenous peroxidase was inactivated with 3% H_2_O_2_ in distilled water for 10 min at RT and rinsing with PBS. Subsequently, 100 µl of streptavidin HRP diluted 1:500 in PBS was added to each tissue section for 30 min at RT in a humid chamber, after rinsing the slides twice by immersing them in PBS for 5 min at RT. Finally, the DAB substrate solution was applied to tissue sections until light brown color development was obtained. The slides were then rinsed with d.H2O several times, counterstained with hematoxylin, dehydrated, and mounted with DPX. A negative control slide was incubated with 100 ul of equilibration buffer and biotinylated dUTP without rTdT enzyme, and a positive control slide was incubated with 100 ul of DNase I buffer-containing 1000 units/ml of DNase I (Promega, USA). Moreover, apoptotic cells were counted in four selected microscopic fields per colon tissue (magnification, 400x) to determine the number of apoptotic cells.

### RNA extraction and cDNA synthesis

Total RNA was extracted from colon cancer tissue using TRIzol reagent (Life Technologies, Carlsbad, Ca, USA) according to the manufacturer`s instructions. The concentration of RNA was measured using NanoDrop (Thermo Fisher Scientific Multiskan GO, Finland). The isolated RNA was reverse transcribed to cDNA using a Reverse Transcription kit (Applied biosystem, Lithuania) according to the procedure supplied by the manufacturer.

### Real time PCR

The expression of cytokines was quantified using Quantifast SYBR green qPCR kit according to the manufacturer`s instructions (Qiagen, USA). The qPCR reaction was started by adding 10 µl 2X Quantifast mix, 1 µl (500 ng/µl) cDNA, 0.8 µl forward, 0.8 µl reverse primers, and 7.4 µl nuclease-free water to PCR tubes with a final volume of 20 µl. All cytokine expression levels were normalized to the GAPDH gene. The primer sequences for different genes under study were obtained from Al-Omari et al. [35] and the sequences were as follows: INF-γ FW: TTCTTCAGCAACAGCAAGGC, RV: TCAGCAGCGACTCCTTTTCC, IL-4 FW: GAAGCCCTACAGACGAGCTCA,RV: ACAGGAGAAGGGACGCCAT, TGF- FW: CCTGCAAGACCATCGACATG,RV:TGTTGTACAAAGC GAGCACC, GAPDH (Housekeeping gene) FW:TGCAGTGCCAGGTGAAAATC,RV:ATCACGTCCTCCATCATCCC.TNF-α (FW: CTACCTTGTTGCCTCCTCTTT, RV: GAGCAGAGGTTCAGTGATGTAG. The PCR conditions were 95 °C initial denaturation for 30 s, followed by 40 cycles of 95 °C denaturation for 10 s, annealing at 60 °C for 20 s, and extension for 20 s at 72 °C using Rotor-Gene Q-QIAGEN (Germany). The relative expressions of cytokine genes were calculated using comparative Ct (2^−ΔΔ*CT*^) analysis methods and assayed as in the equations below. ∆Ct = AVG. Ct (gene of interest)-AVG. Ct (housekeeping gene); ∆∆Ct = ∆Ct (treated sample)-∆Ct (control sample).

### Statistical analysis

Statistical analysis was performed using Sigma Plot 14.0. software package (Systat Software GmbH, Erkrath, Germany). The Student’s t-test was applied to compare two sample means on one variable. When more than two groups were involved in the comparison, one-way ANOVA was used. If the normality test failed, was performed the nonparametric Kruskal–Wallis one-way analysis followed by the Mann–Whitney rank-sum or the Tukey post-hoc test. A p-value below 0.05 was considered significant.

## Results

### AAT therapy lowers disease activity index (DAI) in AOM/DSS mice

The acute colitis-induced CAC model was designed as illustrated in Fig. 1A. After single intraperitoneal AOM administration mice were challenged with DSS (2.5% in the drinking water) for three cycles over a period of ten weeks, and AAT was administered once weekly from the end of week 14 to 18 week. Disease activity index (DAI), including body weight loss, presence of blood in stool, and diarrhea was monitored during the experiment period. Treatment with AAT per se resulted in slightly higher body weight (Fig. 1C) but did not influence colon length (Fig. 1D) and did not induce diarrhea or bleeding as compared to vesicle controls. As expected, AOM/DSS treatment increased the DAI score (Table 1, Fig. 1E). Notably, more AOM/DSS mice, which were treated with AAT, had diarrhea relative to AOM/DSS without AAT therapy (Table 1). While the therapy with AAT did not influence AOM/DSS mice weight, the anal bleeding was reduced, and the length of the colon was higher relative to non-treated mice (Figs. 1B and D).

**Table 1:** Disease activity index (DAI): Number of positive occult blood, diarrhea, and anal bleeding mice for each group

Animal Group	Occult Blood positive mice number (%)	Diarrhea positive mice number (%)	Anal Bleeding positive mice number (%)
Control	0/7 (0%)	0/7(0%)	0/7(0%
AAT	0/7 (0%)	0/7(0%)	0/7(0%)
AOM/DSS	7/9 (77.8%)	4/9 (44.5%)	7/9 (77.8%)
AOM/DSS + AAT	4/7(57.1%)	5/7 (71.4%)	2/7 (28.6%) *

^*^ *p* < 0.05 was considered a significant

### AAT therapy lowers tumor numbers

**Fig. 2:**
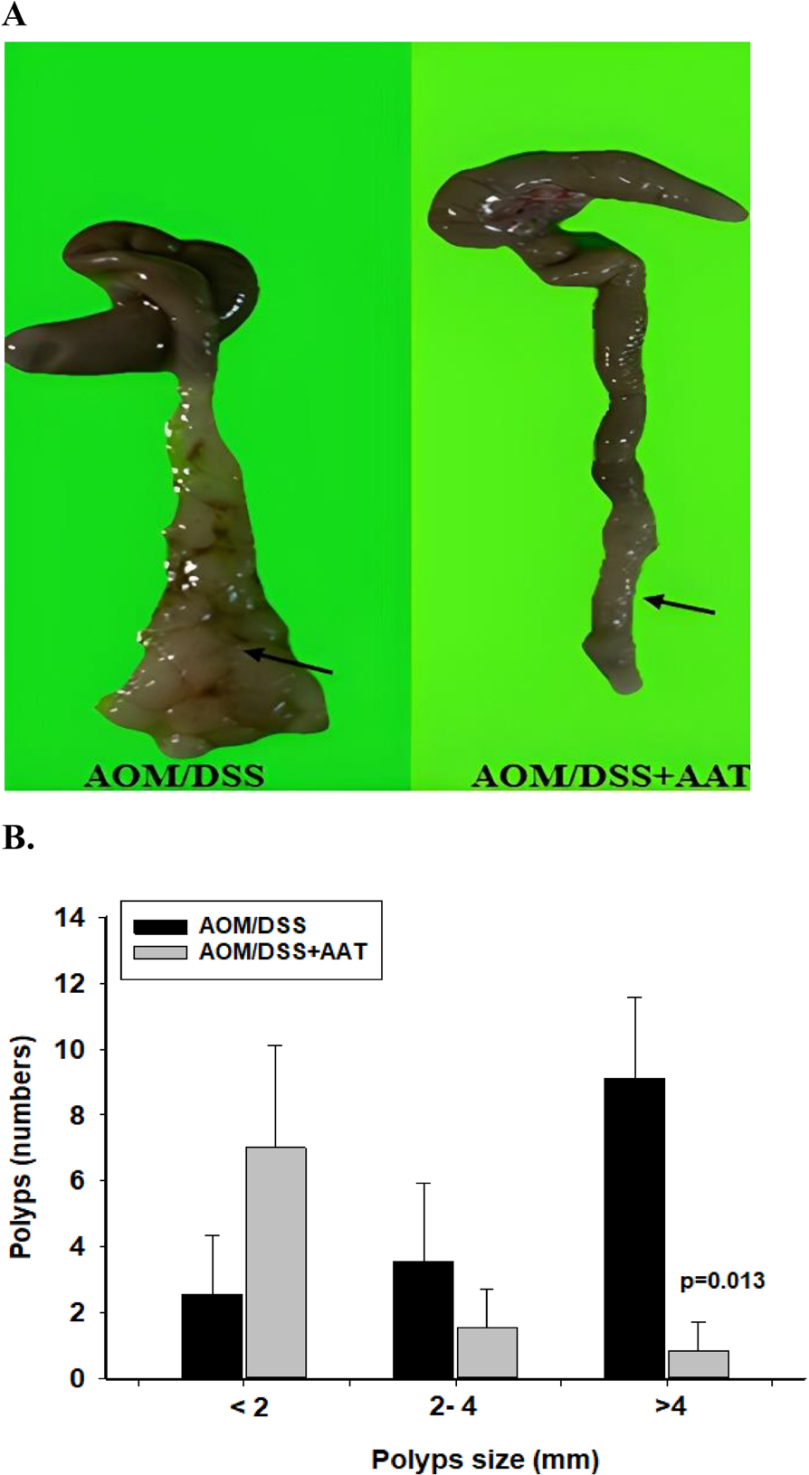
**A** and **B** AAT therapy lowers tumor numbers. **A**. Representative colons recovered from AOM/DSS and AOM/DSS + AAT groups after 18 weeks showing polyps and tumors (blackarrows). **B**. Bars show number of polyps in different size in AOM/DSS and AOM/DSS + AAT group. A value of *p* < 0.05 was considered to indicate a significant difference between groups

As expected, AOM/DSS mice developed multiple tumors, particularly in the middle and the distal colon (Fig. 2A). The macroscopic examinations revealed that the average number of large polyps (above 4 mm) was significantly higher in the AOM/DSS mice than in AOM/DSS treated with AAT [mean (SD): 9 ± 2.2 *vs.* 0.8 ± 0.69, *p* = 0.012]. In general, the AOM/DSS mice had a high number of polyps and tumors of variable sizes and in different locations as compared to those treated with AAT (Fig. 2B).

### AAT therapy reduces tumor progression and intestinal inflammation

**Fig. 3:**
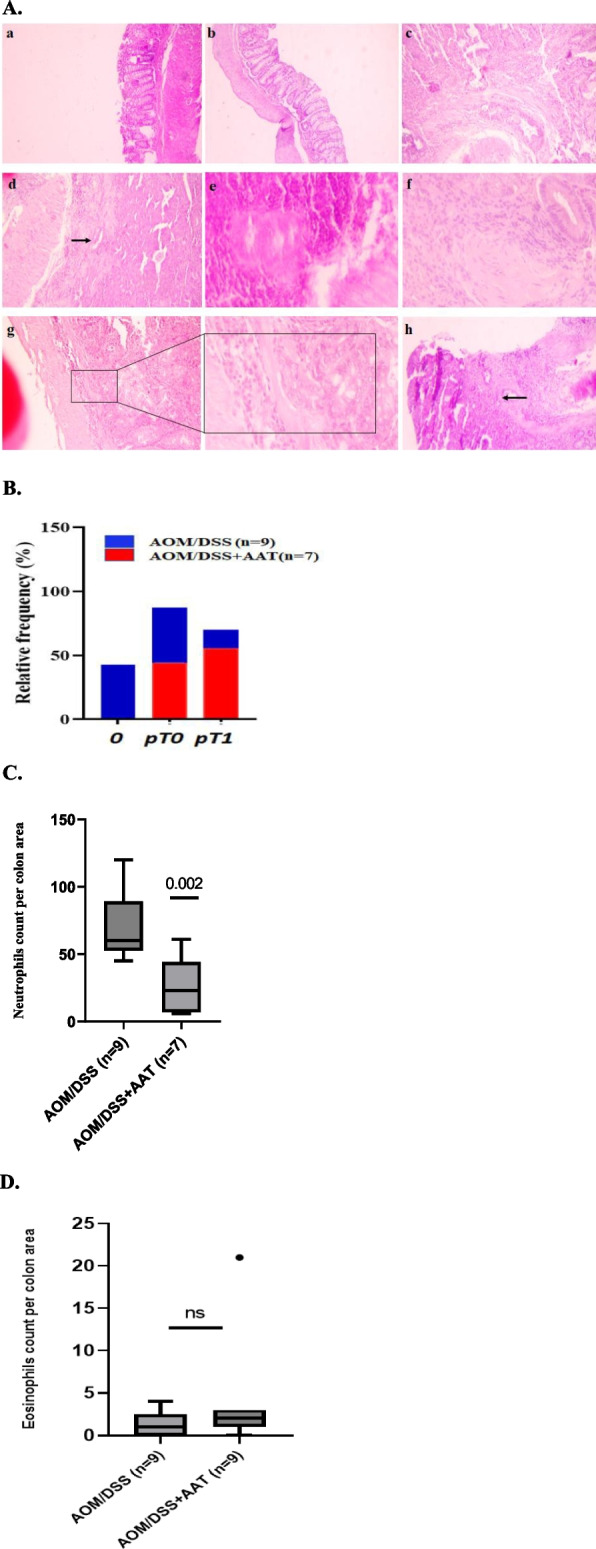
AAT therapy reduces tumor progression and intestinal inflammation. **A.** Representative histopathological images of the colorectal cancer in the mice at week 18**. a**: normal appearance of mouse colon tissue in control group,** b**: control group-treated AAT with normal morphology of colonic wall, **c**: large tumor invasion to *muscularis* mucosa in DSS group, **d**: adenocarcinoma with submucosal invasion, pT1 (*Arrow*) in DSS group, **e**: A peicolic lymph node with adenocarcinoma invasion in DSS group, **f**: colon with perineural invasion in DSS group, **g**: adenocarcinoma invading the muscularis mucosa pT1 in the DSS/AAT group, **h**: adenocarcinoma invading the muscularis mucosa pT1 in DSS/AAT group. Histopathology sections were performed using hematoxylin and eosin staining. **A-f, h** panels were with low power, 100  X magnification and panel (**g**) with low power, 100X, and high power, 400X magnification. **B.** Relative frequency (%) of tumor formation and propagation between groups at week 18. (0) representing an unremarkable colonic mucosa, (pT0) represents tumor intraepithelial or tumor invasion to lamina propria with no extension through muscularis mucosa. (pT1) representing tumor invades muscularis mucosa and submucosa. **C.** Box plots show neutrophils infiltration into colon tissue between groups at week 18. **D.** Box plots show eosinophils infiltration into colon tissue between groups at week 18. Results shown as the mean ± SD. A value of *p* < 0.05 was considered as significant

Next, we performed the histomorphological evaluation of intestinal inflammation, hyperplasia, and tumorigenicity using hematoxylin and eosin (H&E) staining. According to our findings, in AOM/DSS mice cancer cells were found in different parts of the colon including lamina propria (LP, a thin layer of connective tissue) and in *Muscularis* mucosa (MM, a layer of smooth muscle fibers). Three of seven AOM/DSS mice treated with AAT revealed unremarkable colonic mucosa, whereas in the remaining mice, the tumor was found exclusively in the LP and MM regions (Fig. 3A). Furthermore, AOM/DSS mice treated with AAT had less progress in high-grade advanced cancer as compared to the AOM/DSS group. The relative frequency (%) of tumor progression between groups at week 18 was: (0)- representing colonic mucosa 0% in AOM/DSS and 42.2% in AOM/DSS + AAT; (pT0)—tumor intraepithelial or tumor invasion to LP with no extension through MM (AOM/DSS 44% *vs.*0.42.2% AOM/DSS/AAT), and (pT1)- tumor invasion in MM and submucosa (AOM/DSS 55.5% *vs.0.1*4.2% in AOM/DSS/AAT) (Fig. 3B). Histological evaluation of inflammatory cell infiltration into the colon revealed significantly higher neutrophil counts in the AOM/DSS compared to the AOM/DSS/AAT [mean (SD): 70.3 (8.2) *vs.* 26.6 (7.9), *p* < 0.001] (Fig. 3C, S1). The numbers of eosinophils were low and similar in both groups of mice (Fig. 3D, S1).

### Therapy with AAT reduces apoptotic and caspase-3-positive cancer tissue cells

**Fig. 4:**
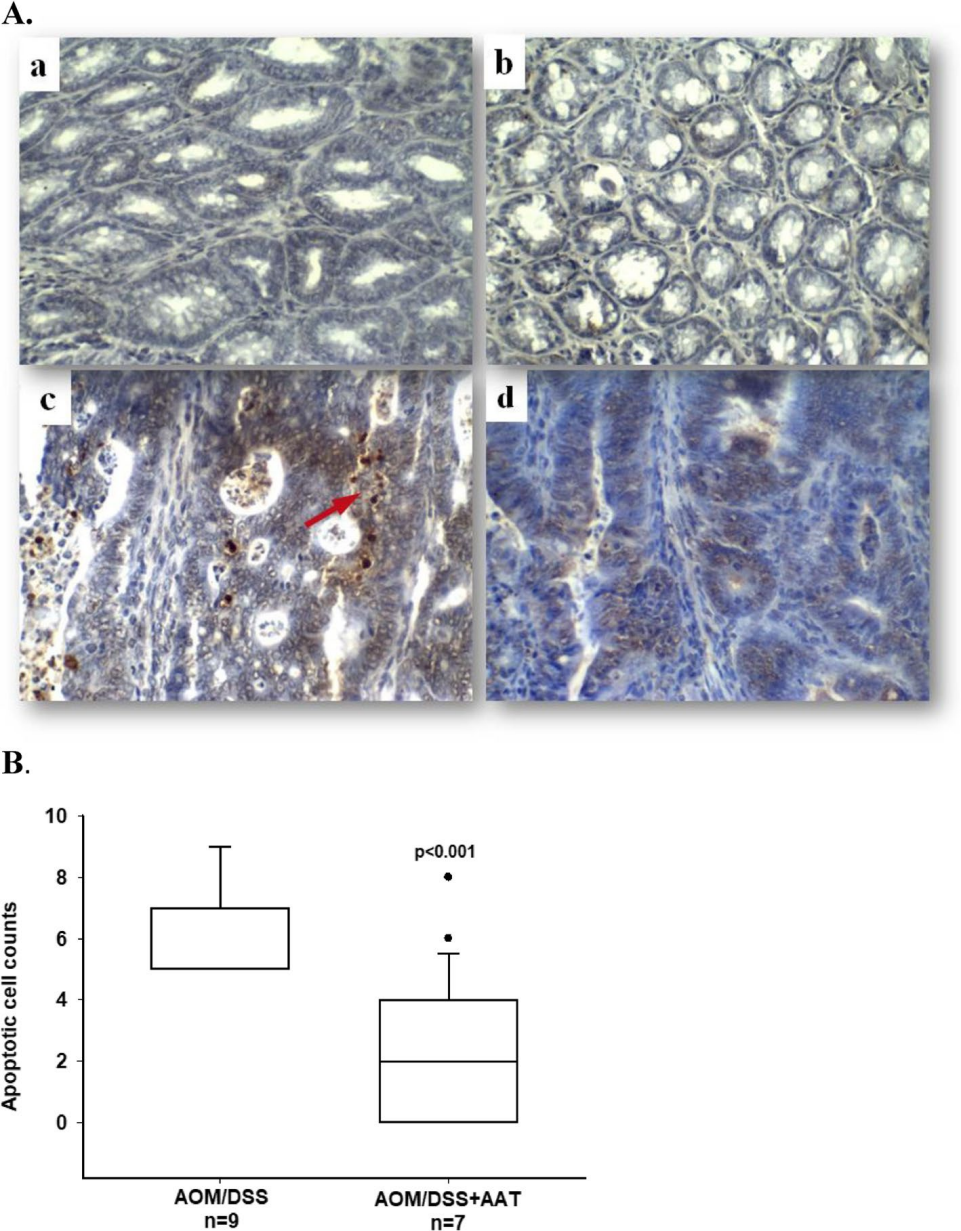
Therapy with AAT reduces apoptotic positive cells in cancer colon tissue. **A**. representative images of cell apoptosis in colon tissues detected by using the TUNEL assay: a. Control; b. AAT; c. AOM/DSS and d. AOM/DSS + AAT. Tissue fields at a magnification of 400 X. Red arrows indicate of apoptotic cells. **B**. Quantitative analysis of TUNEL positive cells. The number of apoptotic cells is shown as box plots. Pairwise multiple comparison (Holm-Sidak method) was applied for statistical analysis. A value of *p* < 0.05 was considered as significant

**Fig. 5:**
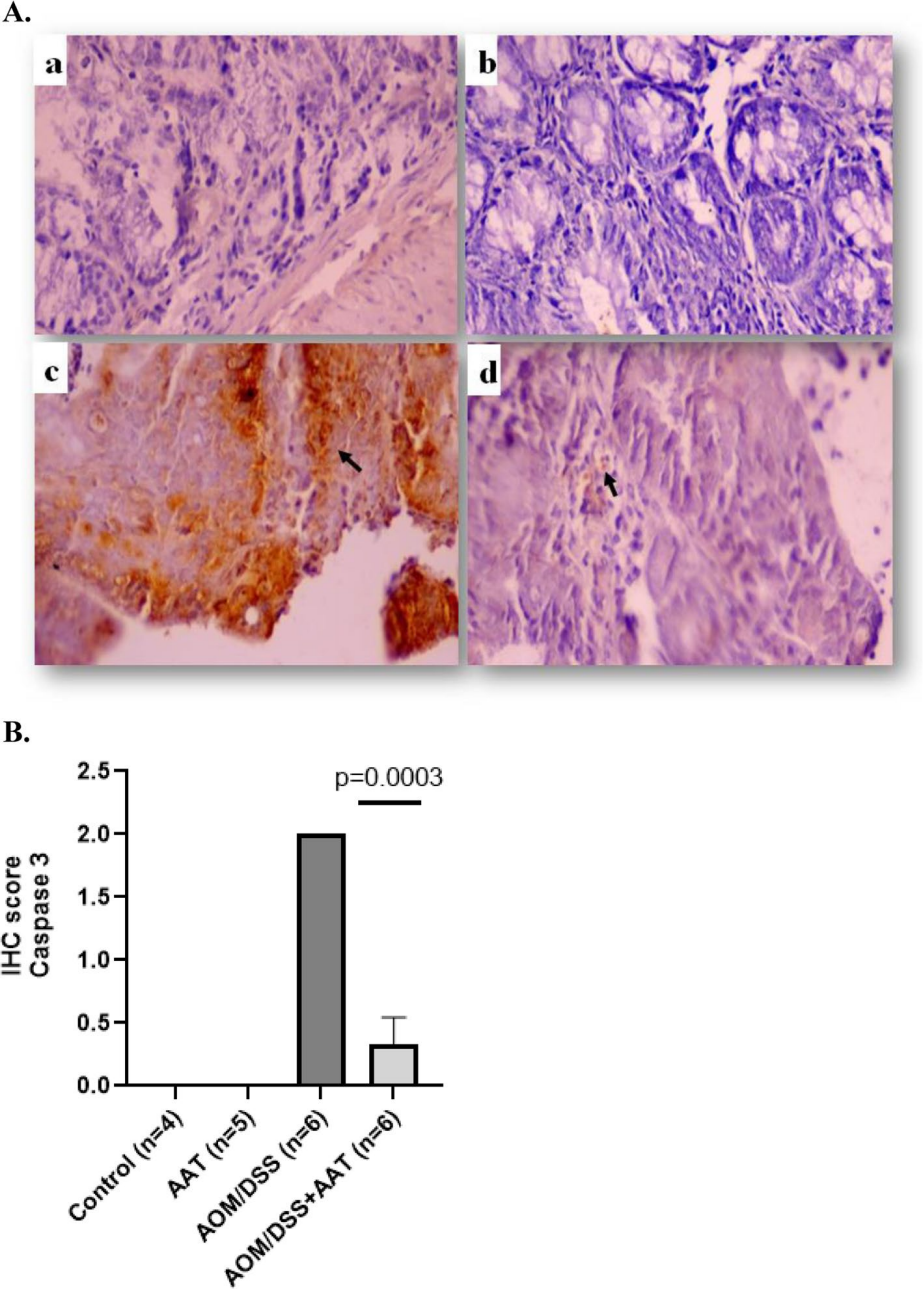
Therapy with AAT reduces caspase-3-positive cells in colon cancer tissue. **A**. Representative images of caspase-3-positive cells in cancer tissue (**a**. Control; **b**. AAT; **c**. AOM/DSS and **d**. AOM/DSS + AAT). Tissue fields are at a magnification of 400X. Black arrows indicate caspase-3-positice areas. **B**. Bars show IHC of caspases-3 score based on the stain intensity (0 = no stain, 1 = weak, 2 = moderate; and 3 = strong). A value of *p* < 0.05 was considered as significant

To investigate putative pathways related to the beneficial effects of AAT therapy, we focused on apoptotic and caspase-3- positive tumor cells in AOM/DSS mice. As shown in Figs. 4A and B, the number of apoptotic cells per analyzed area was significantly lower in AOM/DSS/AAT than in AOM/DSS mice. Remarkably, a strong to moderate positive staining for caspase-3 was found exclusively in the colon cancer tissue cells of AOM/DSS mice whereas significantly lower staining intensity and frequency were observed in AOM/DSS mice treated with AAT. Notably, caspase-3 staining was not detected in the colon cells of the control group treated with AAT, but a weak to moderate staining was detected near the villi surface of the non-treated control mice (Figs. 5A and B).

### Therapy with AAT lowers MMP9 but elevates Granzyme B levels in the colon cancer tissues of AOM/DSS mice

**Fig. 6:**
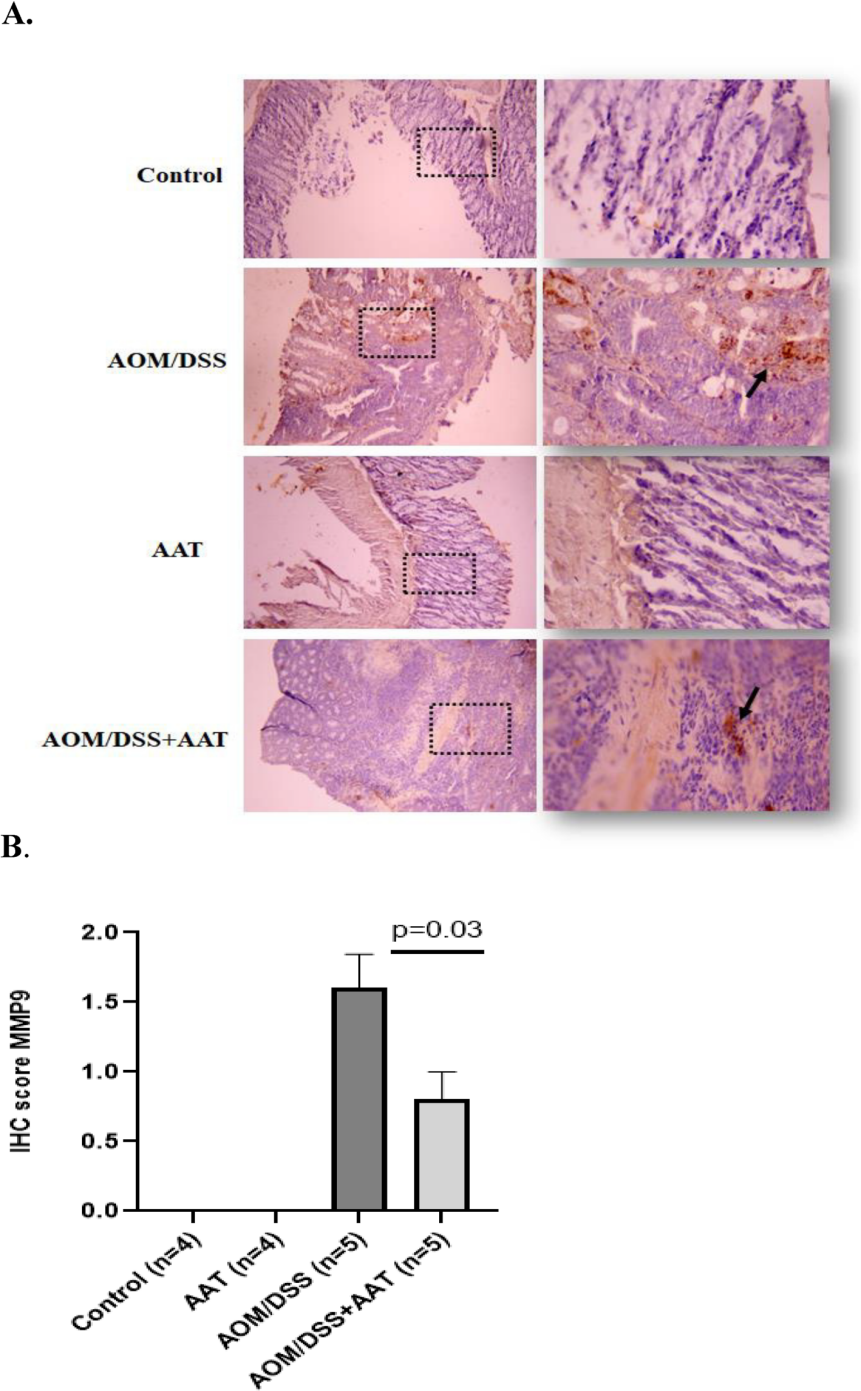
Therapy with AAT lowers MMP-9-positive cells in colon cancer tissue. **A**. Representative images of MMP-9 staining in colon tissue for four experimental groups. The tissue fields at a magnification of 100 × and 400 × respectively. The black arrows indicate areas of MMP-9-positive cells. **B**. Bars show IHC score of MMP-9 based on the stain intensity (0 = no stain, 1 = weak, 2 = moderate; 3 = strong). A value of *p* < 0.05 was considered as significant

**Fig. 7:**
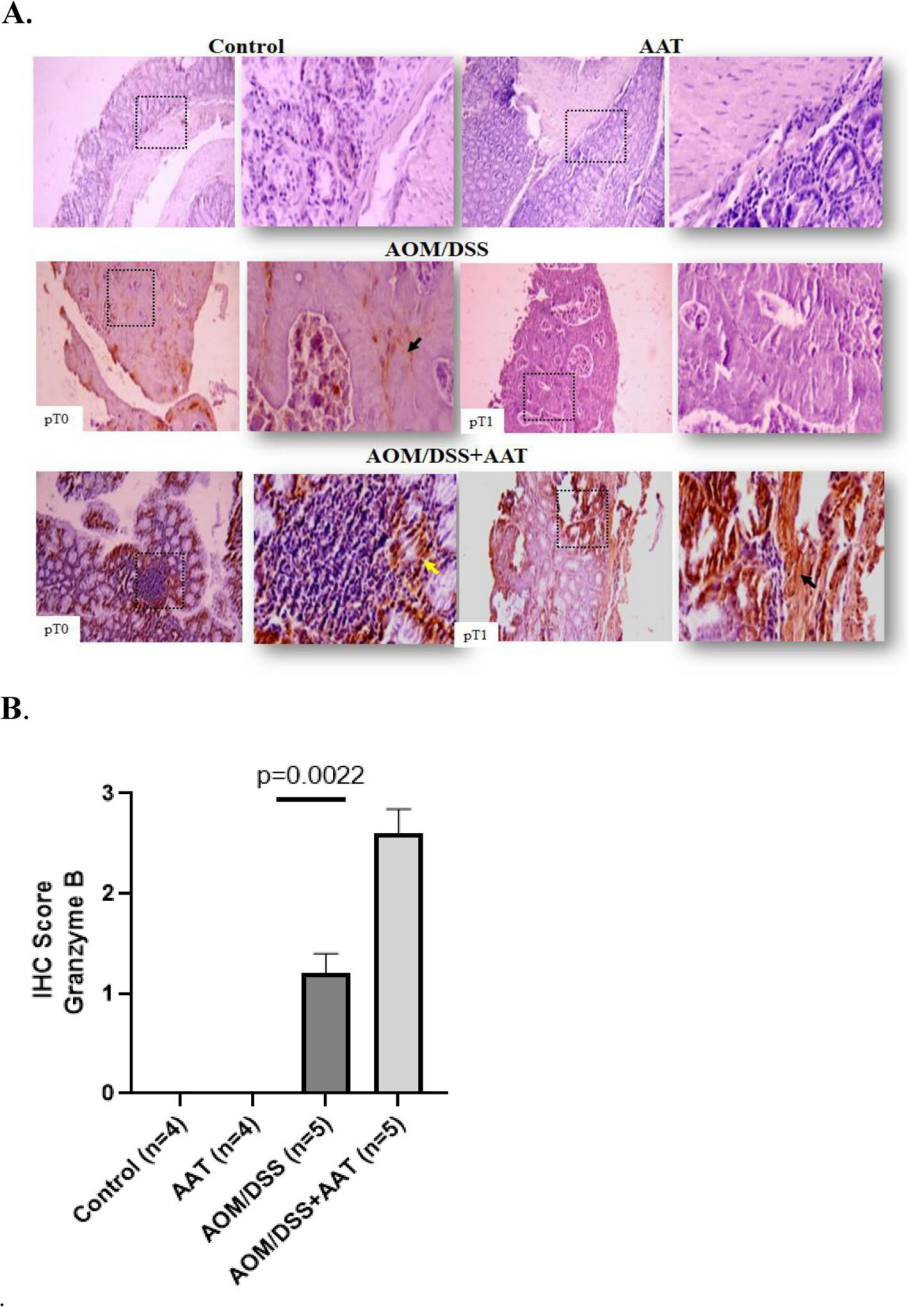
AAT Therapy elevates Granzyme B levels in the colon cancer tissues. **A**. Representative images of Granzyme-B-positive cells in colon of experimental groups (staining were performed in 4 mice per group). pT0 shows tumor invasion to the lamina propria with no extension through the muscularis mucosa; pT1 represents tumor invades the muscularis mucosa and submucosa. The tissue fields at a magnification of 100 X  and 400 X  respectively. The arrows indicate Granzyme-B-positive cell areas. **B**. Bars show IHC of Granzyme B score according to the stain intensity (0 = no stain, 1 = weak, 2 = moderate; and 3 = strong). A value of *p* < 0.05 was considered as significant

Subsequent analysis of MMP-9 and Granzyme-B positive staining in a colon of AOM/DSS mice without and with AAT therapy revealed significantly more MMP9-positive inflammatory cells in the AOM/DSS than in the AOM/DSS treated with AAT (Fig. 6A). Notably, tumor cells were negative for MMP9 staining. Moreover, cells in AAT-treated and non-treated control mice were also negative for MMP9 staining (Figs. 6A and B). Regarding colon cell positivity for Granzyme-B, we found no positively stained cells in AAT-treated and non-treated control mice, whereas colon samples of AOM/DSS + AAT mice with cancer pT0 or pT1 stages were significantly more positive than samples of AOM/DSS mice (Figs. 7A and B).

### Therapy with AAT induces the expression of IL4 and INFG and reduces TNFA in colon cancer tissues of AOM/DSS mice

To evaluate the anti-inflammatory effects of AAT therapy we analyzed the expression of inflammatory cytokines in colon cancer tissues. As shown in Figure S2, AOM/DSS mice treated with AAT show significantly higher *IL4,* but slightly lower *IFNG* and *TNFA* mRNA as compared to AOM/DSS mice.

## Discussion

The extension and duration of colon inflammation are considered highly significant risk factors for the initiation and progression of CRC [36]. Currently, a mouse model of AOM/DSS-induced CRC is one of the most widely used chemically induced models providing important insights into the mechanisms of inflammation-related colon carcinogenesis, drug discoveries, and/or validation of novel therapeutics. An important advantage of this model is a relatively short timeline since tumor development typically occurs within 10 weeks [32]. Moreover, the histopathology of AOM/DSS-induced tumors well recapitulates key features of human colitis-associated cancer [33].

Human AAT is one of the major anti-protease and anti-inflammatory proteins [37]. Therefore, inherited and/or acquired deficiencies of AAT might favor proteolysis and persistent inflammation, which can benefit the initiation of carcinogenesis. In support, previous studies demonstrated a relationship between low levels of AAT and a risk for colorectal cancer [23, 38]. Concomitantly, other studies found that a therapy with AAT attenuates colitis and chronic ileitis through the suppression of cytokine production [39]. These findings led to the idea that commercially available human AAT preparations, designed to treat emphysema patients with inherited AAT deficiency, might be useful beyond inherited deficiency states. In fact, a short while ago Qing Cai, and co-authors reported that CRC development in colitis associated AOM/DSS mouse model is characterized by neutrophilic inflammation, oxidative stress, and increased serine protease activity. Furthermore, the authors demonstrated that augmentation therapy with AAT (commercial preparation, Aralast NP) suppresses inflammatory response and proteolysis and inhibits cancer progression [40].

In keeping with a lately published report, we further confirm the preventive and therapeutic potential of another commercial preparation of human AAT (Prolastin) in the AOM/DSS mouse model. Colon mucosal inflammation, colon length shortening, and body weight loss characterize colitis induced by DSS. According to our results, AAT therapy significantly prevents colon shortening, and anal bleeding, and diminishes histopathological changes in the colon of AOM/DSS. In parallel, therapy with AAT reduced polyp numbers and tumor sizes, which paralleled lowered neutrophil infiltration. Current knowledge suggests that the reduction of tumor number and size can occur after depletion of neutrophils, which massively infiltrate the lamina propria and submucosa during the progression of colitis-associated CRC [41, 42]. Thus, AAT inhibits CAC development, most likely, as a negative regulator of neutrophil recruitment.

It is important to point out that the number of Granzyme B-positive-cancer and inflammatory cells in colon tissue increased upon AAT therapy. Granzyme B is a pro-apoptotic cytotoxin produced and secreted by immune and non-immune cells but also by neoplastic cells, e.g., cancer of the colon [43]. For instance, Salama and co-authors observed that a higher number of tumor-associated Granzyme B-positive cells is associated with better survival among CRC patients [44]. The relationship between Granzyme B expression and tumor stage has also been suggested [45]. Granzyme B can promote cell apoptosis through a direct cleavage and activation of cysteine proteases, specifically caspase-3 [46, 47]. Alternatively, mitochondrial cytochrome release might be the primary mode of Granzyme B-induced apoptosis, and caspase activation is not required for cytochrome release [48]. We did not study cytochrome release in our experimental model, however, the finding that AAT therapy significantly lowers the numbers of tumors and caspase-3-positive cancer cells in the colonic tissues, supports the idea that Granzyme B employs different apoptotic pathways and/or enhances natural cytotoxicity. Notably, tissue-protective role of AAT has previously been related to its property to inhibit caspases, specifically caspase-3 [49].

The excessive activity of matrix metalloproteinases (MMPs), especially MMP9, can directly contribute to cell apoptosis. Moreover, neutrophil MMP9 seems to be important for tumor progression [50]. The therapy with AAT reduced the numbers of MMP9 (gelatinase B)- positive cells in cancer tissues. As already mentioned above, AAT therapy significantly lowered neutrophil infiltration into colons of AOD/DSS mice. Thus, lowered neutrophil counts and concomitants reduction in MMP-9-positive tumor tissue cells together with increased Granzyme B-positive tumor-associated cells, reflect the anti-cancer mechanisms of AAT augmentation therapy.

Cytokines of the intestinal microenvironment dominate immunological responses, and therefore the abnormalities in the expression of cytokines, like IL-4, and IFN-γ, mirror the dysregulation of intestinal immunity associated with pathological processes, including cancer [51]. Moreover, the intrinsic defense cells, especially activated macrophages produce pro-inflammatory cytokines, like TGF-β and TNF-α, which directly or indirectly affect the intestinal epithelial cells [52]. The induction of acute DSS colitis is characterized by extensive epithelial erosion, loss of goblet cells, leukocyte infiltration, and simultaneous increase in expression of the TNF-α and IFN-γ in colon tissues of diseased animals [53]. Interestingly, AAT therapy significantly induced expression of *IL4* and slightly induced *IFNG* but lowered *TNFA* and showed no effect on *TGFB* in colon tumor tissues of AOM/DSS mice. IFN-γ was described as one of the most highly upregulated cytokines in the DSS mouse model of intestinal inflammation [54], which causes a breakdown of the vascular barrier through the disruption of the adherents junction protein VE-cadherin and is a crucial driver of DSS-induced experimental colitis [55]. Indeed, increased *IFNG* mRNA in tumor tissues of AOM/DSS mice receiving AAT might be linked with higher number of mice having diarrhea in this group. This observation remains to be addressed in further studies.

Taken together, our data support beneficial effects of AAT therapy in the CAC mice model, which can be attributed to the reduced neutrophilic inflammation and direct and/or indirect reduction of colon cancer development. It is known that AAT regulates inflammatory responses via both -protease inhibitory and non-inhibitory functions. For example, in vitro findings show that AAT binds to cell surfaces, enters intracellularly via lipid rafts, and promotes a switch from pro-inflammatory to anti-inflammatory pathways. Moreover, AAT can directly interact with inflammatory molecules and abrogate their activities. Among others, AAT can scavenge reactive oxygen species, and interact with free heme, defensins, IL-8, and leukotriene B. It is also worth mentioning, that AAT interacts with lipoproteins and free fatty acids. These pleiotropic properties of AAT provide a rationale for testing AAT therapy outside patients with inherited AAT deficiency. So far, the therapy with AAT showed beneficial effects in experimental models of transplant rejection, ischemia–reperfusion injury, collagen-induced arthritis, graft-versus-host disease, experimental autoimmune encephalomyelitis, preeclampsia, and inflamed pancreatic islets, among others [56–64]. Small clinical trials have been conducted to address the potential benefit of AAT therapy for patients with graft-versus-host disease, acute myocardial infarction**,** and cystic fibrosis [65, 66].

## Conclusion

Newly published data and results presented in this study suggest that AAT therapy expressing anti-inflammatory and anti-protease activities inhibits early progression of colorectal cancer in mice. Hence, commercial preparations of human AAT might be of interest for testing in patient cohorts with IBD-related cancer.

## Supplementary Information

**Additional file 1:** **Figure S1.** Histological evaluation of inflammatory cell infiltration into the colon. Representative histopathological images of the neutrophils infiltration in colon cancer at 18-week: A. AOM/DSS, and B. AOM/DSS+AAT. Representative histopathological images of the eosinophil’s infiltration in colon cancer in the mice at 18-week: C AOM/DSS, and D. AOM/DSS-AAT. Tissue fields at a magnification of 400 X. Arrows indicate inflammatory cells. **Figure S2.** Fold change in expression of *TNFA, INFG, TGFB and IL4* in mouse colon tissues. The expression of *TNFA, INFG, TGFB and IL4* was analyzed by RT-PCR. The mRNA levels were normalized against GAPDH used as a housekeeping gene. The Tukey post-hoc test was used to evaluate the results statistically. A value of *p* < 0.05 was considered a significant.

## Data Availability

All data generated or analysed during this study are included in this published article [and its supplementary information files].
