# Supplementary material for: Beneficial effects of alpha-1 antitrypsin therapy in a mouse model of colitis-associated colon cancer
Source: BMC Cancer. 2023 Aug 2;23:722. doi: 10.1186/s12885-023-11195-5 (PMC10394932; doi:10.1186/s12885-023-11195-5)
Supplement: Supplementary file 1 — Additional file 1: Figure S1. Histological evaluation of inflammatory cell infiltration into the colon. Representative histopathological images of the neutrophils infiltration in colon cancer at 18-week: A. AOM/DSS, and B. AOM/DSS+AAT. Representative histopathological images of the eosinophil’s infiltration in colon cancer in the mice at 18-week: C AOM/DSS, and D. AOM/DSS-AAT. Tissue fields at a magnification of 400 X. Arrows indicate inflammatory cells. Figure S2. Fold change in expression of TNFA, INFG, TGFB and IL4 in mouse colon tissues. The expression of TNFA, INFG, TGFB and IL4 was analyzed by RT-PCR. The mRNA levels were normalized against GAPDH used as a housekeeping gene. The Tukey post-hoc test was used to evaluate the results statistically. A value of p < 0.05 was considered a significant. [file 12885_2023_11195_MOESM1_ESM.docx]

**Supplementary Figure 1. Histological evaluation of neutrophils (A and B) and eosinophils (C and D).**

**
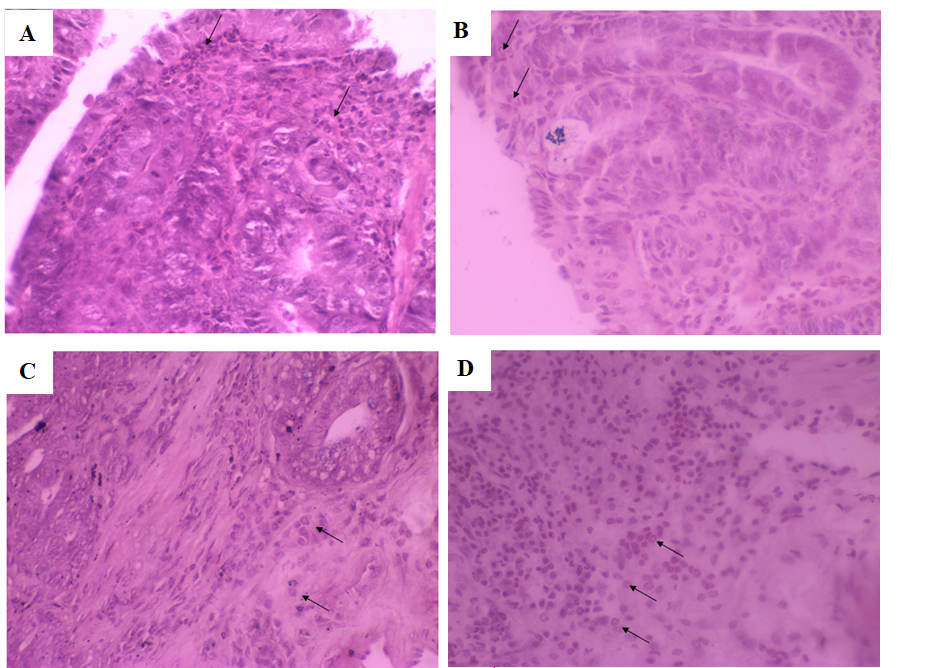
**

**Figure S1.** Histological evaluation of inflammatory cell infiltration into the colon.

Representative histopathological images of the neutrophil infiltration in colon cancer at 18-week**: A.**AOM/DSS, and **B**. AOM/DSS+AAT. Representative histopathological images of the eosinophil’s infiltration in colon cancer in the mice at 18-week: **C** AOM/DSS, and **D.** AOM/DSS-AAT. Tissue fields at a magnification of 400 x. Arrows indicate inflammatory cells.

**Supplementary Figure 2.**


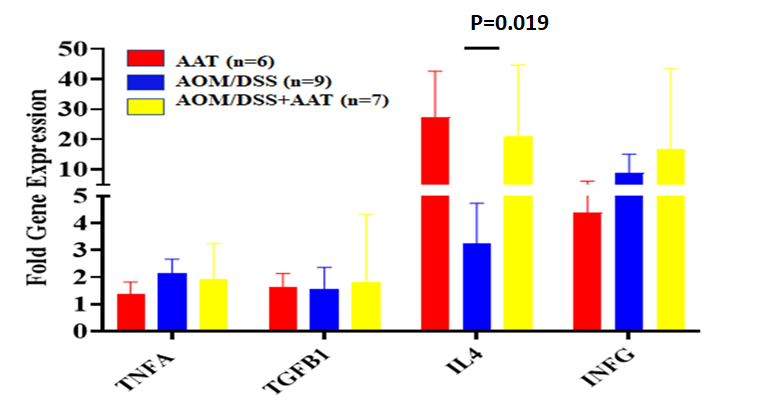


**Figure S2.** Fold change in expression of *TNFA*, *INFG, TGFB and IL4* in mouse colon tissues. The expression of *TNFA*, *INFG, TGFB and IL4* was analyzed by RT-PCR. The mRNA levels were normalized against GAPDH used as a housekeeping gene. The Tukey post-hoc test was used to evaluate the results statistically. A value of p < 0.05 was considered a significant
